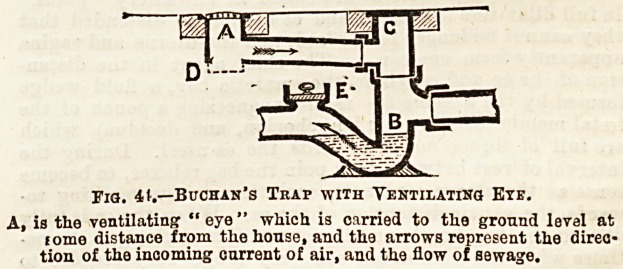# "The Hospital" Nursing Mirror

**Published:** 1896-09-26

**Authors:** 


### "The Hospital" Nursing Mirror.

Being the Extra Nursing Supplement of "The Hospital" Newspaper.

[Contributions for this Supplement should be addressed to the Editor, The Hospital, 428, Strand, London, W.C., and should have the word "Nursing" plainly written in left-hand top corner of the envelope.]

### News from the Nursing World.

### GOD SAVE THE QUEEN!

This week marks the extension of the reign of Queen Victoria beyond that of any other English Sovereign. Her Majesty has no more loyal subjects than the nurses whose profession has been called into being during this long and splendid reign, and they will one and all join heartily in the wish that for many years still may our "Sovereign Lady" live to rule over us. It is for her womanly sympathy and kindly interest in the sorrows and joys of her people that our Queen is specially beloved; and it is largely due to the encouragement ever given by her to every effort for the mitigation of suffering that, when the glories of the present reign come to be reckoned up, not the least among them will be the spread of skilled nursing for rich and poor, not only throughout England, but in the Colonies and in India, where women indeed have reason to bless the name of Victoria.

### THE BRITISH ASSOCIATION AT LIVERPOOL.

Members of the medical profession in Liverpool have celebrated Sir Joseph Lister's presidency of the British Asssociation and the visit of the Association to their town by a variety of entertainments. On Saturday evening a banquet was given in the Philharmonic Hall, and amongst the audience in the galleries and on the platform were numbers of nurses in the uniforms of their various institutions. In his speech, Sir Joseph Lister commented on the excellent arrangement for the training of nurses which he was glad to know existed in Liverpool.

### NURSES' HOURS IN AMERICA.

The annual report of the Brooklyn Homœopathic Hospital contains details of the nurses' day's work in the wards, which may be interesting to English nurses. The routine varies in many particulars from that which is familiar to workers in English hospitals. The nurses rise at half-past six, some going to breakfast at seven, others going first to the wards for half-an-hour, an arrangement which we may congratulate ourselves does not obtain in this country. Then the patients' breakfasts are served, and the wards made ready for the visits of the doctors. At twelve and half-past twelve the nurses go to dinner, the patients having their meal at one p.m. From either one to three, three to five, or six to eight, each nurse goes off duty, having also every week a half-day from two to eight p.m., during which time she "is expected to rest, to write up her lectures, or to go out of doors." At five o'clock the nurses have "supper," the patients having theirs at six. The day nurses' work is over at eight p.m., and the wards put in readiness for the night nurses. Prayers are held in the nurses' sitting-room at eight, and afterwards there is a lecture by one of the doctors, or a lesson from the superintendent. At ten o'clock day nurses must be in bed. We hope they have some refreshment between that early supper and bed. Night nurses are on duty from eight p.m. to seven a.m.

### MEDICO-PSYCHOLOGICAL ASSOCIATION.

We are asked to state that the next examination of this association for the certificate in nursing and attending on the insane will be held on Monday, November 2nd next, October 5th being the last day upon which candidates can enter their names. Candidates should obtain from the registrar, Dr. Spence, Burntwood Asylum, near Lichfield, a schedule to be filled up, signed, and returned to him at least four weeks before the date of the examination.

### A LADY HOSPITAL SECRETARY.

Mrs. T. E. Cope has been appointed to the secretaryship of the Royal Eye Hospital, Southwark. We believe it is the first time that a woman has been chosen to fill the important post of a London hospital secretary. Mrs. Cope is the lady who was sent out by Government in charge of the royal and other exhibits shown at the Chicago Exhibition by the Women's Royal Commission under H.R.H. Princess Christian.

### "NURSES' CO-OPERATION," LEICESTER.

We have received particulars of a newly-organised nursing institution in Leicester, "established to secure to nurses the full remuneration for their work," and undertaking to supply the public with fully-trained medical, surgical, mental, maternity, fever, and children's nurses. The matron is Mrs. J. Foster, who sends rules, stating, "the management is vested in a General Committee, half of which are appointed an Executive Committee, to be elected every six months." It is not explained, however, of whom these committees are composed, the proportion of nurse representatives upon them, or whether this is a public or private venture? It is of the first importance to nurses to know this, and we shall be glad to receive precise information on the points.

### OFFICIAL DELAYS.

The Christchurch Guardians are showing a commendable anxiety to provide suitable accommodation for their infirmary nurses, and are complaining bitterly that they cannot get the plans for new buildings promptly passed by the Local Government Board. The nurses have an energetic champion in Mr. Druitt. This gentleman, in proposing at a recent meeting of the Board that the Local Government Board be pressed for a speedy reply, urged that better accommodation should be found for the nurses as soon as possible "in order that the guardians might escape the disgrace of using their most faithful servants in the way they were now used." Another guardian corroborated Mr. Druitt's remarks, adding that it "was very wrong for the nurses who have to look after the sick poor to have worse accommodation than the sick poor themselves," an incontrovertable statement, which met with general approval. The guardians of this infirmary wisely obtain their nurses through the Workhouse Infirmary Nursing Association.

### FIVE YEARS' GOOD SERVICE.

The people of Alfreton, Derbyshire, are regretting the temporary loss of their Queen's nurse, who, after five years' work among them is obliged to take a six months' rest. Nurse Rowley has made for herself many friends in Alfreton, and amongst these a movement is on foot to present her with some gift in recognition of her valued services to the sick poor amongst whom she has laboured. We hope Nurse Rowley will find herself quite strong again after a long holiday.

### NURSING IN DENMARK.

The calling of a nurse is held in high esteem in Denmark, and nurses are comparatively well paid, both in hospital and private work, especially in Copenhagen. In the hospitals the nurses have a right to a pension when incapacitated from further work by age or illness. There are many district nursing societies, supported mainly by grants from the Government.

### ON DUTY NIGHT AND DAY.

Truly the proceedings of boards of guardians are instructive to read of. The guardians of an East Anglian Union have been amazed at the sudden resignation and departure of a recently engaged assistant nurse, who left behind her a letter objecting to the double duties of night and day nurse being centred in herself. "Mr. Mobbs," reports the local paper, "did not think they could expect the nurses to work both night and day. The master replied that if required the nurses had to be at work at night, but *as a rule* (the italics are ours) on those occasions they arranged for a rest during the day"! And yet, though similar notions on the subject of nurses' powers of endurance and the needs of sick people unfortunately are common in many quarters, guardians are surprised that good nurses do not answer their advertisements!

### MISPLACED CONSIDERATION.

"It is really a most respectable village," remarked the churchwarden; "there is practically no drunkenness, and the people come to church very regularly." "Yes," replied the rector, "they are excellent folks, and amazingly charitable. They always give silver when there's a collection." Encouraged by this liberality, the churchwarden proposed to introduce a Hospital Sunday collection, and this was duly announced. To the rector's chagrin, the contributions proved exceedingly limited. "I cannot understand this," he said; "our local hospital has always been deservedly popular, and its collecting-boxes are very well filled." "Yes," explained the churchwarden, "but they take coppers there. Your congregation say they could not offer you less than silver, and the threepennies have run short this week in the village!"

### LONDON SCHOOL OF MEDICINE FOR WOMEN.

The winter session of the London School of Medicine for Women opens on October 1st, and is to be inaugurated by a conversazione and musical entertainment at the Royal Free Hospital. For the first time, we believe, in the history of this school, the opening address will be delivered at the hospital. Mr. Boyce Barrow is the selected lecturer, and the proceedings will begin at half-past eight p.m.

### CONVENTION OF NURSES IN THE UNITED STATES.

On September 2nd a convention of nurses met at Manhattan Beach Hotel to consider the constitution of an "Articulated Association of Graduate Nurses" in the United States and in Canada. The details of the scheme, which has been slowly forming for some years, will be given next week.

### A GLASGOW NURSE.

Miss Samuel, assistant matron at the Western Infirmary, Glasgow, is giving up her post at the end of this month, and leaving Scotland for Central Africa, where she is to organise and take charge of the Mission Hospital at Blantyre, about to be opened there by the Church of Scotland. Miss Samuel has been connected with the Western Infirmary for over nine years, during which time she has gained the respect and esteem of all the staff. The nurses have presented her with a parting gift in the shape of a handsome gold bangle, and she will be followed to her new work with many cordial good wishes.

### A DISTRICT NURSING ASSOCIATION FOR CAMDEN TOWN.

District nurses are gradually extending their useful work through town and country, but many are the areas still uncared for in this respect. In Camden Town and its neighbourhood, including the parish of St. Mark's, Regent's Park, there is a district containing some 45,000 inhabitants, nearly all belonging to the very poor, where there is as yet no resident district nurse. The Rev. E. B. Penfold, vicar of St. Michael's, Camden Town, Rural Dean of St. Pancras, is warmly advocating a scheme for supplying this large district with a Nursing Association, and a resolution to the same effect has been passed by the North St. Pancras Branch of the Charity Organisation Society. It is impossible to overestimate the good influence exercised in the homes of the poor by nurses skilled in their work and trained not only to nurse the sick, but also to teach the people habits of cleanliness and a better knowledge of sanitation. Contributions in aid of the proposed association should be sent to Rowland Humphreys, Esq., 27, Fellows Road, N.W.

### SHORT ITEMS.

Miss M. H. Carter, late "Sister Albert," of St. Thomas's Hospital, was married to Mr. McKellar on Saturday last. The wedding took place at Hornsea, Yorkshire.—It has been decided by the Vestry of St. George-the-Martyr, Southwark, to appoint a lady as sanitary inspector in that district. Twenty-one applications have been received for the post.—A fête recently held in aid of the South Devon and East Cornwall Hospital, Plymouth, and the Royal Albert Hospital, Devonport, resulted in a profit of £120. The Earl of Mount Edgcumbe threw open his beautiful park, as well as the gardens and conservatories, on this occasion. Unfortunately the weather was stormy.—Mrs. Garrett Anderson, Dean of the London School of Medicine for Women, has set the seal of her approval on bicycling, and is said to have become herself an enthusiastic "wheelwoman."—Two Indian lady medical students have lately qualified for the Edinburgh L.R.C.P. diploma. One is a daughter of the late member for Finsbury, Miss Dadhabai Naoroji; the other bears the name of Miss Manek Atmaram Pandurang.—At Torquay a bicycle gymkhana, inaugurated by the Dowager Lady Haldon, has been lately held for the benefit of the Nurses' Institute.—We have to acknowledge the receipt of 30s., for the Hospital Convalescent Fund, from "An Evangelist in connection with the Methodist church."

### Hygiene: For Nurses.

By John Glaister, M.D., F.F.P.S.G., D.P.H.Camb., Professor of Forensic Medicine and Public Health, St. Mungo's College, Glasgow, &c.

### XXV.—HOUSE DRAINAGE (*continued*).—TRAPS; SIPHONING OF TRAPS. FORMS OF TRAPS.

Traps may fail in their purpose from one of three main causes, viz.:—(1) from "siphon" action, (2) from momentum or (3) from evaporation of water-seal. What is meant by "siphoning" of a trap? The action of the syphon having already been explained, little difficulty should be experienced in the comprehension of this. Let us consider the problem in its practical application to a soil-stack into which a series of closet-branch pipes enter at different levels, as in Fig. 41. In the first place, let it be clearly understood, that "siphoning" most readily happens where the traps of the soil-pipe and sanitary fittings are unventilated. Therefore, in the diagram, the soil-pipe is not carried above the eaves, the traps of the various closets are unventilated, as is also the trap of the soil-pipe at its junction with the house-drain. Let us suppose, to begin with, that the trap of each closet is properly filled with water, thus trapping the atmosphere of soil-pipe from that of house-interior, and that the soil-pipe and branch-pipes are full of air. Between the trap of closet I, and the unventilated trap of soil-pipe C, there is, therefore, an intervening column of air locked between these two water-sealed points. When closet I. is used, what happens is what is represented in the figure The descending column of water (D) rushes into the soil-pipe, and, like the piston of a pump, compresses the atmospheric column already mentioned, which is water-locked at C. This atmospheric column, therefore, in front of the descending water column is at a greater pressure than that of the outside air, and the air column behind the water is at less pressure. When the water descends to D, and more particularly when it reaches the junctions of the branch-pipes from closets II. and III. (F and G) the difference of pressure is at once noticed in the water of the traps of these closets. And since the pressure at F and G is less than that on the surface of the water in the traps II. and III., the greater pressure drives the water out of the traps into the soil-pipe after the descending column of water, and thus these traps are unlocked, or unsealed, or "siphoned," and are consequently left empty. Suppose, for example, the ordinary atmospheric pressure being 14·7 lbs. per square inch on the surface of the water in the traps II. and III., that the area of water in each trap be equal to 48 square inches, and the reduction of pressure within the soil-pipe during the action of the flush be ½ lb. per square inch, then the total pressure, by difference, on the surface water of the traps will be 48÷2=24 lbs., and this increase of pressure drives the water of the trap into the soil-pipe in its effort to establish atmospheric equilibrium in the soil-pipe atmosphere. This phenomenon of "siphoning" takes place, under such circumstances, even more readily when a bath is being emptied high up in the waste-pipe or soil-stack, for then the pipe is running "full bore" with water. When traps are siphoned a straight path for the foul air of the soil-pipe is opened into the house interior.

Fig. 41.

Momentum is the second common cause of rendering a trap useless. When the descending column of water in the soil-pipe reaches the unventilated trap, C, it has attained considerable velocity, which is not reduced much by the basin of the trap; consequently, when the last portion of the column reaches the trap it is in a highly-agitated condition, and since a body moving at considerable velocity is difficult to bring to a state of rest, the whole column is likely to be swept through the trap, which is thus left partially or entirely empty.

The third cause is disuse of the trap, during which time evaporation of the water-seal happens. This is apt to occur in town houses which are unoccupied wholly or in part for periods during summer, or, indeed, at any time. By reason of this evaporation the atmosphere of soil-pipe and house becomes continuous, and not only foul gases, but also micro-organisms, owing to the dry condition of the pipes, find an easy access into the house.

Fig. 42.

Traps may also be rendered inoperative from being blocked with solid deposit owing to imperfect "setting" or to imperfect water-flushing, or to insufficient fall in house-drain.

Fig. 43.—Buchan's Intercepting Ventilating Trap.

From a knowledge of these difficulties experience has suggested remedies. For example, free ventilation of soil-pipe and all traps connected therewith will prevent siphoning. Any temporary disturbance in the equilibrium of the atmosphere of soil-pipe is thus quickly and safely righted. The remedy for disuse is obvious. The means of ventilating the soil-pipe having been already discussed, it will be enough now to consider the means of ventilating the traps of the branch fittings. Fig. 42 shows the position of the ventilating pipe in an ordinary trap of a lead pipe. It is always placed on the "crown" of the trap—on the soil-pipe-side of the trap;

Fig. 44.—Buchan's Trap with Ventilating Eye.

A, is the ventilating "eye" which is carried to the ground level at some distance from the house, and the arrows represent the direction of the incoming current of air, and the flow of sewage.

where a series of such ventilating pipes exist, they are united either to a separate ventilating main pipe, or, separately, into the soil-pipe at some distance above the entrance of each branch, this distance being regulated (1) by the maximum flush of water which can enter the soil-pipe at any time, and (2) the length of soil-pipe which that amount of water will occupy. Thus, one gallon of water fills a pipe of 4 in. diameter to a height of 22 in., and a pipe of 4½ in. to nearly 17½ in. If the maximum flush be three gallons, and the pipe 4 in. in diameter, then $22 \times 3 = 66$ in. would be the height above the entrance of the branch pipe at which the ventilating pipe of the trap of that branch should enter the soil-pipe. In large traps which are placed in the ground at the termination of the soil-pipe or waste-pipe, ventilation is attained by a special opening in the trap which reaches the surface of the ground, and which is usually covered by a grating. Fig. 43 shows the essential points of a good trap; and Fig. 44 the arrangement for carrying the ventilation opening at ground-level some distance from the house wall.

The essential points of a good trap are these, viz.: (1) it should be of a self-cleansing shape; (2) it should be ventilated, and thus not easily unlocked from any cause; (3) it should possess a sufficient water-seal—not less than 2½ in.; (4) it should not be liable to "silt"; and (5) it should have an opening for cleansing purposes. In Fig. 43 the water-seal is shown by the dotted lines, and, Z, represents the tongue or "dip" of the trap. The precise form of the trap is regulated by the special function it is intended to have, and the forms now in use are so varied that space forbids their consideration.

### Midwifery Papers.

### V.—LABOUR—MECHANISM—STAGES—DELAYS.

In the mechanism of labour there are three important points to be considered: (1) The body (the fœtus) to be expelled; (2) the passage (the pelvis) through which the fœtus passes; (3) the expelling force (uterine and muscular contractions) which drives the body through the passage. These three factors must bear a certain relation to each other in order that their work may be successfully performed; the fœtus must be of the size and shape to fit the pelvis through which it has to pass; the construction of the pelvis must admit of the free and yet gradual descent of the fœtus; and the muscular contractions of the uterus must be of sufficient force to expel the child. The absence of any of these conditions prevents natural labour taking place.

The child's head is most frequently the "presenting part," or the part which descends first through the pelvis, and is born first. It enters the pelvis at the brim in the oblique diameter, and in its descent through the pelvic cavity it accommodates itself to the pelvic curves by a series of movements. The first movement, flexion, takes place at the brim, when the child's chin presses forward on its chest, thus bringing the occiput (or back of the head) lower in the pelvis. The gradual descent of the head and rotation forwards of the occiput then takes place until the floor of the pelvis is reached; the head then lies in the antero-posterior diameter of the pelvic outlet, the forehead lying in the hollow of the sacrum and the occiput pressing under the pubic arch. The next movement, extension of the chin from the chest, is aided by the pressure of the occiput on the pubic arch, which acts as a fulcrum, and brings the child's head and face over the perineum, and the head is born. The last movement, restitution, consists in the child's face turning up to the mother's thigh, and is caused by the rotation of the shoulders into the antero-posterior diameter, preparatory to being expelled. All these movements (1) Flexion, (2) Descent, (3) Rotation, (4) Extension, (5) Restitution must be performed before the child can be born. In labour there are three stages, each marked by the achievement of a certain event. The first stage dates from the earliest regular contractions of the uterus to the full dilatation of the os-uteri. These uterine contractions are called in midwifery "pains." In full dilatation the cervix and os become so distended that they cannot be longer recognized, and the uterus and vagina apparently form one canal. The chief agent in the distension of the os and cervix is the amniotic bag, a fluid wedge formed by the uterine contractions squeezing a pouch of the fœtal membranes (the amnion, chorion, and decidua), which are full of liquor amnii towards the os-uteri. During the interval of rest between each pain the bag relaxes, to become tense as the uterus contracts, each time doing something towards the gentle distension of the os. When the os is fully dilated the bag bursts and liquor amnii is discharged. Sometimes when the membranes are very tough they require to be artificially ruptured, and this is done by carefully nicking them with the finger nail or the point of a silver probe. The full dilatation of the os marks the outset of the second stage of labour, which is completed by the expulsion of the fœtus. After the discharge of the liquor amnii the uterus contracts more powerfully on the child, and "bearing down" pains set in. The intervals between the pains are shorter, the presenting part advances more quickly during a pain, to recede slightly during the interval. When the presenting part presses on the perineum each pain is accompanied by a strong impulse to "bear down," and the mother is able to do something to assist the birth. The perineum gradually stretches and becomes very thin, and when the presenting part appears at the vulva the perineum is stretched to its utmost capacity, and during a strong pain it recedes over the head, and the child is born. During this extreme tension of the perineum lacerations often occur, and it is just then that special care should be taken.

The third stage of labour is completed by the discharge of the placenta. The strong uterine contractions gradually detach the placenta from the uterine surface, and it is generally expelled about twenty minutes or half an hour after the birth of the child. The uterine contractions close the large vessels which are open and bleeding over the placental site. After the expulsion of the placenta the uterus contracts into a firm, hard ball, the size of a cricket ball. This condition disappears when the lochia (uterine discharge) sets in. Delays in labour may occasionally arise from the presence of certain conditions on the part of the mother or of the child. On the mother's part the conditions may be: (1) Uterine inertia; (2) rigidity of cervix, vagina, and perineum; (3) early rupture of membranes; (4) prolapse of anterior vaginal wall or lip of os; (5) pendulous abdomen; (6) small vagina; (7) abnormal growth in vagina; (8) ankylosed coccyx; (9) contracted pelvis. I will give a few hints as to the nurse's treatment of each. (1) Uterine inertia is caused by (*a*) debility or morbid condition of uterine wall which often occurs in a uterus which has borne many children, (*b*) pressure of loaded bladder or rectum; (*c*) excess of liquor amnii; and (*d*) the increased distension of the uterus caused by twin pregnancy. The treatment to follow in the case of (*a*) is to give rest and nourishment, and after a suitable time has elapsed, should the labour be still retarded, to send for a doctor; in (*b*) to pass the catheter or give an enema; in (*c*) and (*d*) to puncture the membranes. For (2) rigidity of soft parts give a warm douche and anoint the parts with warm olive oil. (3) In early rupture of the membranes leave the labour as long as possible to nature, carefully conserving the strength of the patient, but send at once for a doctor if there are any signs of exhaustion. (4) In an interval between the "pains" push up the prolapsed vaginal wall or lip of the os. (5) Apply a tight binder, especially if the child lies over the pubes, as it sometimes does in a pendulous abdomen. (6) In a small vagina leave the labour without interference so long as some progress is being made, but should the pains be strong, with no advance of the presenting part, send at once for a medical man. In the cases of (7), (8), and (9) send at once for a medical man.

On the part of the child some delays are caused by (1) breech presentation; (2) large child; (3) ascites; (4) hydrocephalus; (5) abnormal presentations; (6) non-flexion and rotation; (7) dorsal displacement of the arm. The treatment in (1) and (2) is to leave the labour to nature so long as there is no exhaustion and no hæmorrhage. In all the other cases, directly they are recognised, summon a doctor at once, for they cannot be properly treated by a midwife.

### Nursing in Alexandria.

A Correspondent writes: The Greek Hospital at which I was a sister for two years stands in a broad road between the town and Ramlah Station, and is a beautiful large white stone building. It has white marble pillars and brown outside shutters to the French windows and stands in an extensive garden bright with Eastern flowers, trees, and shrubs. It is surrounded by a high stone wall and entered by very handsome iron gates opened by the Greek gate porter, who speaks, besides his own tongue, Italian, Arabic, and French, and also a smattering of English. Passing through the gates and crossing a square four marble steps lead into an arcade formed of three fine marble arches connecting two pavilions. On the right are the dispensary, laboratory, out-patients' department, the gynæcological examination-room and one male ward. Upstairs there is a female ward, operating theatre, and a smaller one where only laparotomies are performed. A second large ward has not as yet been in requisition. The doctors' dressing-room, lavatories, and a fair sized room lies on the left. In the latter, which contains every surgical appliance, and has glass shelves fixed to the walls holding glass barrels of disinfectants with tubes leading down to the various dressing tables, all the dressings are done for surgical cases who can be moved out of bed. This with the bath-rooms and ward kitchens attached to each large ward completes one side of the hospital. Before crossing the balcony where the patients are allowed to smoke and walk and from which they have a charming sea-view, there is a large square space cut off the corridor by glass doors, and in winter all patients allowed up take their meals here. In the summer they use the balcony at the side of each large ward as a refectory. On the left are the director's offices, doctors' office, sisters' sitting-room, stores, linen-room, bed-rooms, dining-rooms, kitchens, and bath-rooms, &c. Upstairs is the male medical and surgical ward. All the floors and stairs in the hospital are either white marble or mottled stone, washed daily with a solution of carbolic. The walls are composition, in appearance like white marble; these are also washed every six weeks or two months. There are 140 beds; five large wards contain 16 beds each, and three double and three single-bedded rooms are attached to each large ward. The former are for second-class patients, who pay five francs per day; the latter for first-class ones, who pay ten francs per day. In the large wards third-class patients pay three francs per day. There are isolation wards some distance away from the main building for small-pox, phthisis, diphtheria, erysipelas, and lunatics. There being no asylum near Alexandria, nor, I believe, in Egypt, mental cases are admitted if the doctors think they will benefit by a course of treatment. One gentleman, a native of Alexandria, a fairly harmless lunatic, was there for five years, finally dying from epilepsy. Antiseptic measures are very strictly carried out through the whole hospital. The tops of the lockers and tables are of marble, and are washed daily with a solution of carbolic; the window-ledges, which are about two feet deep, bedsteads, and doors, with paraffin, which helps to keep mosquitos away. All dressings and instruments are kept in glass air-tight jars or nickel boxes, and are sterilised daily, the instruments being boiled for half an hour in a solution of soda or carbolic before each operation. Before an operation—of which there are a great number—the patient having had his bath, the surface around the s at of operation is first is brushed with a nail-brush and oil, soap, and carbolic, then carbolic only and dried, then with spirits of wine, and then sublimate 1-1,000. Over the whole surface is laid a compress of sterilised absorbent wool soaked in 1-1,000 sublimate, with a piece of sterilised batiste rinsed through the same solution, which is kept in place by a bandage also rinsed through sublimate. The patient has a sterilised shirt put on, and is enveloped in a sterilised sheet. He remains in his bed, which is freshly made up with entirely clean things, until the time of the operation, when he is taken in the same sheet and a clean blanket to the theatre, where the compress is removed and a second washing, like the previous one, takes place on the operation-table just before the operation. All sponges and compresses, which are made of absorbent wool and gauze, are boiled and kept in sublimate, and are fresh for every operation. In the theatre all tables are covered with glass; the operation-table is of glass with an iron framework; shelves are also of glass. The instruments, which are all the latest and best, are in one piece, thus do not suffer by boiling, and are kept in a glass cupboard. The operator changes entirely, and puts on sterilised things, and a long mackintosh cloak with short sleeves, which fastens behind, over which he wears a batiste apron previously rinsed out in sublimate. All who assist at the operations wear brown holland sterilised gowns with short sleeves, and after once having cleansed their hands in the same way as the patient was washed are not allowed to touch anything unsteriiised. The patients are held, if required, by the Greek or Arab nurses, who do not help otherwise in the theatre except by emptying basins and replenishing lotions, &c. As regards medical work we had bilious typhoid, which very much resembles yellow fever, and is fatal in the majority of cases; enteric fever, dysentery, continuous fever, eye diseases, especially ophthalmia, and gastric cases filling the beds. Accidents are very few in proportion, most occurring on the Greek Easter and New Year's Eve, when fire-works and shooting are incessant. Maternity cases are taken at the Greek Hospital, and the English Government sends all the soldiers' wives in. The bedsteads are of iron, and have large spring mattresses, which can be lifted off. The mattresses, pillows, and bolsters are stuffed with a kind of white flock, which is cooler than feathers. The first and second class beds have mosquito curtains. The food for the patients and staff is very good and abundant. All patients on full diet are allowed a Greek wine, much like claret. The wards, corridors, and staircases are lofty, wide, cool, and elegant, and decorated with lovely plants. In the corridors, at a certain distance apart, there are trays with carbolic 1-20, which are replenished daily. The windows and doors are kept open winter and summer, and it is very pretty to see the birds flying fearlessly in and out of the wards. Some of the sisters' bed-rooms are next to the wards, but others are away from them. Their sitting-room is beautiful with handsomely-painted walls and ceiling, and divans all around. The summer uniform is fine white longcloth; the winter one navy blue washing material with white spots in the mornings, and navy blue cashmere in the afternoons, and white caps and aprons. The nurses wear brown holland and white caps and aprons, their caps being a different shape. The sisters are supposed to have four hours off duty daily, though this cannot always be managed. The sister of the male medical ward also supervises the infectious block. Before entering she robes herself in a sterilized holland long-sleeved cloak. Small-pox is of a very malignant type, and diphtheria appears in its worst form. Anti-toxin is used with great success, three horses being kept for the purpose of supplying serum to all parts of Egypt. The hospital, built and maintained by the Greek community specially for Greek patients, is open to all nationalities and diseases on payment, but certain exceptions are made for urgent Greek cases, which are admitted gratis. Patients who are not allowed to get up are permitted during certain hours to smoke in bed; and even the females sometimes indulge in it.

The head doctor, Dr. Zencarol, a Greek, is a fatherly and most kind and energetic gentleman. He is recognised as the most clever operator in the East. People come to him from all parts of Egypt. Troops of patients wait hours for him daily and besiege his carriage when it enters the hospital gates. If he has been away for a day or two he is followed by quite a crowd on leaving the hospital. There are five other doctors, all of whom are Greeks except one Italian, who has a practice in the town, but comes to help with the numerous dressings. Greek, Italian, French, German, Arabic, and of course English are spoken in the hospital by different members of the staff. There are five English sisters, and last year we had also two English nurses. The servants are men, either Greeks or Arabs, and they have male nurses, except the two women employed in the female ward and the two English nurses. It is difficult to secure good workers among the natives, but when found they are, as a rule, clever and reliable. Three of the sisters and one nurse were ill while I was there, and no expense nor trouble was thought too great to be lavished in each individual case. One can but admire and esteem the Greeks of Alexandria. I have nursed for over eleven years, and have nowhere met with more kindness than I experienced in Alexandria.

### Jottings from the Punjab.

Perhaps some of the readers of The Hospital may be interested to know something about the places where the sisters belonging to the Indian Nursing Service are stationed. The largest and on that account the most interesting station is Rawalpindi. Here there are quarters for four or five sisters, and here, too, Miss Loch, the lady superintendent of the Indian Nursing Service, has resided since her arrival in this country with her first batch of nurses in 1888. Rawalpindi is one of the most important military stations in India, having barracks which will contain over 4,000 European troops. The ordinary garrison consists of four British and three native regiments. The regiments stationed there this year are the 4th Dragoon Guards, the Gordon Highlanders, the King's Own Scottish Borderers, and the Rifle Brigade, so amongst all these soldiers there is a good deal of nursing work to be done, as can well be imagined. The cantonment is situated south of the native town and is separated from it by the river Leh. The most charming spot to be found in Rawalpindi is the park, where pleasant drives and rides can be taken in the early morning or in the cool of the evening. The district is intersected by mountain ranges on either side, and smaller hills run through the valleys, making the surface of the country very diversified. On one side are the Murree Hills, which form part of the great Himalayan range. Murree is only forty miles distant from Rawalpindi, and large numbers of persons visit this sanatorium during the hot weather, and the invalid troops are sent to the station hospital to convalesce. At Murree too is a hospital for sick officers, and the nursing sisters working there belong to Lady Roberts' nursing staff. Rawalpindi has two rainy seasons, the first from January to March, and the second from July to August; the winter weather is cold, but the summer heats are great. In that part of the hospital where the sisters work, the wards are mostly filled with patients suffering from enteric fever — generally young recruits whose fresh young blood seems more ready to catch any poisonous germs that may be hovering about, than that of older men. When the heat is very oppressive during the summer months, there are often cases of heat apoplexy. Last year there were eighteen or more cases admitted. At such times no ceremony can be used; it is a case of life or death. The sister on duty calls for ice, and mussacks of cold water are poured over the unfortunate Tommy, and every means available used to reduce his bodily heat to a normal level. There are three sisters attached to the hospital, and it is part of their work to train the soldier orderlies to nurse their comrades. During times of emergency and during any great press of work, the sisters are assisted by the lady superintendent. Most of the soldiers appreciate the kindness and good nursing they receive from the sisters here, who undoubtedly exercise a good influence over the men.

The military hospital at Sialkote, where the nursing sisters belonging to Lady Roberts' staff work during the winter months, is situated at the extreme end of one side of the cantonment. The hospital is capable of holding two hundred beds; it has two large blocks, each block containing four wards. At the end of the sisters' ward is a special ward for sick prisoners, for at Sialkote there is a large military prison for British soldiers, who are sent there from all parts of the Punjab and northern frontier stations. Sir George White, the present commander-in-chief in India, was much pleased when he inspected this hospital last year, and said that he had been told that it was "the best managed military hospital in India, bar one," but that he thought it was "second to none." The sisters' quarters are at the entrance of the hospital grounds. They are small, but cosy and comfortable for the winter, and within an easy distance of the hospital. Sialkote is a fairly large military station, having two British regiments (the 11th Hussars and the Royal Scots Fusiliers) stationed there, besides a battery of artillery and two native regiments. In 1857 the native troops quartered at Sialkote mutinied and laid siege to the ancient fort of Salwan, which stands on an eminence in the centre of the city, where all the Europeans had taken refuge, and where they made a gallant defence. The cemetery at the foot of the stronghold contains the remains of the victims killed at the time of the siege. There is a handsome English church and also a Roman Catholic and Presbyterian church, the latter was erected in memory of the Rev. William Hunter, a Scotch minister, who with his wife and child was killed by two native sepoys while trying to make his escape to Lahore at the time of the Mutiny. The district of Sialkote is irrigated by various streams, and is highly cultivated and very fertile; the gardens in the cantonments are large and well kept, and flowers and fruits, such as oranges and lemons, grow in great abundance. Jammu, twenty-two miles from Sialkote, is the chief town of the Kashmir State; the Maharaja of Kashmir has many palaces here, and one was erected for the Prince of Wales on his Royal Highness's visit in 1876. In one of the suburbs of Sialkote is located a small colony of workmen, whose inlaid work in gold and iron has acquired considerable reputation; this art was formerly applied to the adornment of armour, such as swords, daggers, shields, and helmets, but is now confined to peaceful objects, caskets, vases, buckles, combs, brooches, bracelets, and the like, and I believe specimens may be seen by any of The Hospital readers in London who visit the India and Ceylon Exhibition now open.

### The Book World for Women and Nurses.

### The Leisure Hour.

A delightful article on "Johnson in Eighteenth Century Oxford" appears in the September number of this magazine. The frontispiece of the article is an illustration of the beautiful and quaint Kettel Hall, now a private residence. "The Round Towers of Ireland" will attract many readers, who will find much of interest in this number of the magazine.

### Nurses in 1896—Their Quarters, Hours, and Food.

### UNIVERSITY COLLEGE HOSPITAL.

### I.—Terms of Training.

The nursing at the North London or University College Ho pital is under the direction of the All Saints' Sisterhood, the head of each ward being a "Sister" in the religious as well as in the hospital meaning of the word. Probationers pay for their training, except in a few instances when by special arrangement with Sister Cecilia, the Sister Superior, the fees are remitted. At the present time there are said to be no free vacancies for two years. Probationers are admitted for training between the ages of twenty-four and thirty years, and pay for one year £30, exclusive of washing. Certificates are given only on completion of three years' training, and probationers who wish to qualify for this pay £20 for the second and £15 for the third year, the payments being made half-yearly in advance. Probationers are expected to attend the courses of lectures given by members of the medical and surgical staff, and the classes held by the sisters on the lectures, and they are required to satisfactorily pass examinations on the subjects, both theoretical and practical, which have formed the three years' instruction before being considered eligible for a certificate. Nurses are not appointed on the permanent staff until they have completed their three years' training.

### II.—Hours of Work and Times Off Duty.

The day staff begin work in the wards at a later hour than is customary at most hospitals. Probationers and nurses are called at seven a.m.; the former go to their wards at half-past eight, the latter a quarter of an hour later, leaving them for the night at nine p.m. Probationers are allowed two hours off duty only three times a week. At the end of six months, if they give satisfaction, they are called "assistant nurses," and have two hours off duty twice a week and one free evening from six to ten p.m. By permission of the Sister Superior, on these evenings they are allowed to sleep out of the hospital, returning in time for duty next morning. Nurses "can have permission from the sister of their wards to be absent for two hours at a time up to eight p.m.," after which hour leave must be obtained from the Sister Superior. Night nurses are on duty from ten minutes to nine p.m. to nine a.m. They must "never have less than six hours in bed," and may go out every day either in the morning or the evening. If the former, they must be in by a quarter past twelve; if the latter, they must not get up before four p.m. No whole days off duty are given. Nurses are on night duty for one month at a time. The Sisters, as belonging to a religious order, have their hours arranged in accordance with its rules. The nursing staff at University College Hospital, it will be seen from the hours quoted above, have an appallingly limited off-duty time, the probationers being in the wards on four days in the week for eleven hours, subtracting an hour and a-half for meals, while to none of the staff are accorded the usual hospital "day off." They are, however, able to enjoy a long night's rest, having to be in bed by half-past ten p.m., while they do not get up till seven a.m. Night nurses can also secure ample time for sleep. A month's holiday is given each year.

### III.—Meals.

The day nurses' breakfast is at a quarter to eight a.m., dinners at half-past twelve and five minutes past one, tea at four p.m., and supper at nine. Night nurses have supper or "breakfast" at eight p.m., and dinner at nine a.m. If they go out in the morning, and are hungry before going to bed, they are at liberty at all times to ask the housekeeper for lunch. The night nurses' night meal is the only one for which "allowances" of food are served out, and for this each nurse is provided with a sort of small luncheon basket which she carries away from the evening meal packed with the cold meat, bacon, or eggs, or whatever is provided for the night's refreshment. Tea, coffee, sugar, and butter is served twice a week, and kept in a cupboard in the ward or in the refrigerator. The general dietary varies little from that supplied in most hospitals. A meat breakfast; joints, vegetables, and puddings for dinners; and soup, fish, meat, or cheese and puddings for supper. Coffee, tea, and beer are provided for these meals. Tea is taken to the nurses on duty in the wards.

### IV.—Salaries and Uniform.

As already stated, probationers pay for their training at University, and during the first six months also provide their uniform, which has to be that worn by the nurses. Afterwards indoor uniform is provided, print dresses, aprons, and caps, to the value of about £6 a year. They are required to wear the outdoor uniform of the hospital, and to provide it for themselves, at a certain "hospital" reduction. After probationers become assistant nurses the expense of washing their uniform dresses is defrayed by the hospital; their other washing they pay for themselves. When appointed on the permanent staff, nurses are required to be willing "to work either in the hospital or on the private staff." We understand from Sister Cecilia that the two staffs are not interchangeable, though in the intervals of their cases the private nurses are glad to work in the wards and so keep up their hospital experience. On the permanent hospital staff nurses receive £25 the first two years, afterwards £30. If incapacitated by illness or old age University nurses receive a pension and are provided for. Nurses needing a long rest receive full pay for the first three months, half pay for a second period of three months, and are provided for at the Cottage at Eastbourne in connection with the All Saints' Sisterhood, or elsewhere. In cases of illness or over fatigue nurses are sent to the Cottage free of expense. Staff nurses are provided with outdoor as well as indoor uniform.

### V.—Nurses' Quarters.

The accommodation allotted to the University nurses is very poor and inadequate. It is satisfactory to know that plans for a new and splendid hospital, in which are included those for a new nurses' home in accordance with modern requirements, are already approved. At present the nurses are lodged in two or three old houses adjoining the hospital, from which they will soon be removed to other temporary quarters, while the gradual demolition and rebuilding of the old premises is taking place. Senior nurses have single separate bed-rooms, probationers sleep two or three in a room, divided by curtains. There is a pleasant little sitting-room with a piano, but the dining-room in the basement is a most dreary apartment. The sisters have a community room for their use, and sleep in cubicles. Considering that the training at University is paid for by the probationers, the accommodation given them so far is certainly deplorable, and, taken in conjunction with the scanty off-duty time allowed, leaves a large margin for reform. The provision of a proper nurses' home we hope will be one of the first steps taken in the much needed re-constitution of University College Hospital.

### Everybody's Opinion.

[Correspondence on all subjects is invited, but we cannot in any way be responsible for the opinions expressed by our correspondents. No communications can be entertained if the name and address of the correspondent is not given, or unless one side of the paper only be written on.]

### DISTRICT NURSES AND INFECTIOUS DISEASES.

"A District Nurse" writes: Thank you very much for answering my question so fully, but I think you have made the mistake of thinking that I act as midwife in my district. We do not go to] midwifery cases, except occasionally to *after* confinement cases which may not be straightforward. These do not happen very often, and this, I think, makes a material difference in the case of the puerperal fever. I should be glad to have the opinion of a "Superintendent of District Nurses" on the typhoid question.

### Appointments.

### MATRONS.

New Brighton Convalescent Home for Women and Children.—Miss Emma Dunn has] been selected from amongst 268 applicants to fill the post of Lady Superintendent at this institution. Miss Dunn trained at Charing Cross Hospital in 1883, where she afterwards worked as ward sister. She acted temporarily as matron at the Bristol General Hospital, and was then appointed successively chief staff nurse and assistant matron at the Midland Counties Hospital for Chronic Diseases; matron's *locum tenens* at the Sister Dora Hospital, Milford; and matron at Caldecote House Convalescent Hospital, Bushey Heath, having held this latter post for the last eight years. We wish Miss Dunn every success in her new work.

Stroud General Hospital.—Miss Charlotte Garwood has been appointed to fill the position of Matron at this hospital. She was trained at the Leeds General Infirmary, and for the past eight years has been matron of Mr. Jessop's private hospital at Leeds.

Sheffield Union Infirmary.—Mrs. Amelia C. Lawson has been appointed Superintendent at this infirmary. She was trained at St. Mary's Hospital, Manchester, where she also held the posts of staff nurse and sister, afterwards working as maternity charge nurse and night superintendent at the Sheffield Union Infirmary.

### Minor Appointments.

Oporto, Spain.—Miss F. Irene James has been appointed to the post of nurse to the English residents in Oporto. Miss James was trained at the Evelina Hospital for Children and at University College Hospital in general nursing, and has also had experience in maternity and fever work. For the last two winters she has been doing private nursing in Florence.

Emily Bolitho Home, Penzance.—Nurse Lucy Adkins and Nurse Gertrude Brocklehurst have been appointed to take charge of the Emily Bolitho Home, and the district work in connection with this institution, in Penzance. Both nurses were trained at the Leicester Infirmary, and have since been doing district work in Cornwall. Nurse Lucy Adkins also trained at the York Road Lying-in Hospital, and holds the L.O.S. diploma.

General Infirmary, Sheffield.—Miss Evelyn M. Drinkwater has been appointed Sister of the male medical wards at this hospital. She was trained for three years at Salford Royal Hospital, Manchester, where she afterwards held the post of staff nurse.

Skipton Sick Poor Nursing Association, Yorks.—Miss Mary Rudd has been appointed Senior Nurse for this Association. She was trained at the London Hospital, and was on the private staff of that institution for one year. Since then Miss Rudd has worked as district nurse in Perth, N.B., for three years, and at St. Andrew's, Penrith, Cumberland, for four years, while for the last two years she has been nursing amongst the poor of Wallsend-on-Tyne.

### Notes and Queries.

The contents of the Editor's Letter-box have now reached such unwieldy proportions that it has become necessary to establish a hard and fast rule regarding Answers to Correspondents. In future, all questions requiring replies will continue to be answered in this column without any fee. If an answer is required by letter, a fee of half-a-crown must be enclosed with the note containing the enquiry. We are always pleased to help our numerous correspondents to the fullest extent, and we can trust them to sympathise in the overwhelming amount of writing which makes the new rules a necessity. Every communication must be accompanied by the writer's name and address, otherwise it will receive no attention.

### Queries.

(187) *Training.*—Will you kindly tell me of a hospital where paying probationers are admitted at the age of 23?—*J. E. T.*

(188) *Nursing Books.*—Please tell me of a good book on nursing for a nurse?—*M. F. H.*

(189) *Home Wanted.*—Can you tell me a home where a man of 78 with symptoms of brain softening could be received?—*Sister Grace.*

(190) *Swedish Massage.*—Where is this taught in London?—*Nurse.*

(191) *Cottage Nursing Association.*—How can I get into one of these associations?—*Subscriber.*

(192) *Nurses' Handbook.*—Please tell me of an up-to-date handbook on the details of nursing asepticism at operations?—*Hospital Nurse.*

(193) *Male Nurses.*—Information wanted about the training of male nurses.—*A. M. G., Brighton.*

(194) *Leper Stations.*—Are English nurses ever sent out to nurse at any of the leper stations, and where could information on the subject be obtained?—*Nurse Ruth.*

(195) *Nursing in India.*—I want to join a private nursing institution in India. I have lived in India and speak Hindustani fluently, and my qualifications are one year's general training in a Scotch hospital, three years at a private nursing institute, and a certificate for monthly nursing.—*Nurse Marion.*

(196) *Nauheim Treatment.*—Where can I get a book on this treatment?—*Nurse B.*

(197) *Quarantine and Fever Nursing.*—Is there any Home where nurses can be taken in after nursing infectious cases? Also please tell me how one could get employment as a fever nurse working on my own account.—*Enquirer.*

(198) *Epilepsy.*—Can you tell me of a hospital where a slight case of epilepsy would be admitted?—*Nurse Annie.*

### Answers.

(187) *Training* (*J. E. T.*).—Do you want to know of London or provincial hospitals? You will find many of both mentioned in "How to Become a Nurse" (Scientific Press, 428, Strand), where probationers are eligible at the age mentioned. Write to the matrons.

(188) *Nursing Books* (*M. F. H.*).—"Nursing: its Theory and Practice," by Dr. Percy Lewis, and "Nursing," by Miss Hampton, both published by the Scientific Press, 428, Strand, W.C.; also "Lectures on General Nursing," by Miss Lückes (Kegan Paul, Trench, and Co.).

(189) *Home Wanted* (*Sister Grace*).—You will find complete lists of homes and institutions in "Hospitals and Charities" (Scientific Press, 428, Strand), with particulars of payments. A few paying patients are received at the Hospital for Epilepsy and Paralysis, Portland Terrace, Regent's Park, N.W. You might write for particulars, and possibly the Secretary would be able to tell you of some suitable home, should the case not be eligible for admission there.

(190) *Swedish Massage.*—We shall be happy to reply to "Nurse" if she will send her full name and address in compliance with our rules.

(191) *Cottage Nursing Associations* (*Subscriber*).—The hon. secretary of the Ockley Nursing Association is Mrs. Henry Lee Steere, The Cottage, Ockley. Why do you not go in for full training if you wish to become a nurse? Women are wanted to train for infirmary nursing.

(192) *Nurse's Handbook* (*Hospital Nurse*).—You have not complied with our printed rule, and enclose neither name nor address.

(193) *Male Nurses* (*A. M. G., Brighton*).—There is no training school for male nurses in England as yet. A certain number of men are trained at the National Hospital for Paralysed and Epileptic, Queen Square, Bloomsbury. Perhaps the secretary would be able to help you with advice.

(194) *Leper Stations* (*Nurse Ruth*).—Dr. P. S. Abraham, 2, Henrietta Street, Cavendish Square, might be able to give you the information you require.

(195) *Nursing in India* (*Nurse Marion*).—There is the Up country Nursing Association for Europeans in India (hon. secretary, Major-General J. Bonus, R.E., The Cedars, Strawberry Hill), to which you might apply. A register of applications is kept in connection with this association. Lady Roberts' nurses are chosen by herself, and appointment on her staff can be only through a personal introduction. We believe Mrs. Nisbet, Matron of the Madras General Hospital, might be able to give you information.

(196) *Nauheim Treatment* (*Nurse B*).—Read the articles on the Schott Treatment, which appeared in The Hospital for October 19th, 1895, and January 11th, 1896. There is a pamphlet on this subject, price 6d., by Dr. Greene, published by the Scientific Press, 428, Strand.

(197) *Quarantine and Fever Nursing* (*Enquirer*).—We believe that Miss C. J. Wood, Nurses' Hostel, 27, Percy Street, Tottenham Court Road, W.C., takes nurses for disinfection and quarantine. Write to her, enclosing stamped envelope for reply. (2) Your query is somewhat vague. Do you mean in London? It is never wise to start private nursing unless you have a good and certain prospect of work. You do not say what your training has been.

(198) *Epilepsy* (*Nurse Annie*).—You do not say if the patient in question is male or female, a child or an adult, nor whether payment can be made. There is the Epilepsy Colony at Chalfont St. Peter's, Bucks, for men (offices, 12, Buckingham Street, Strand); and for women, the Meath Home of Comfort, Westbrook, Godalming. Payment has to be made in both cases. There are also the hospitals in London for Epilepsy and Paralysis, Queen's Square, W.C., and Portland Terrace, Regent's Park.

## Figures and Tables

**Fig. 41. f1:**
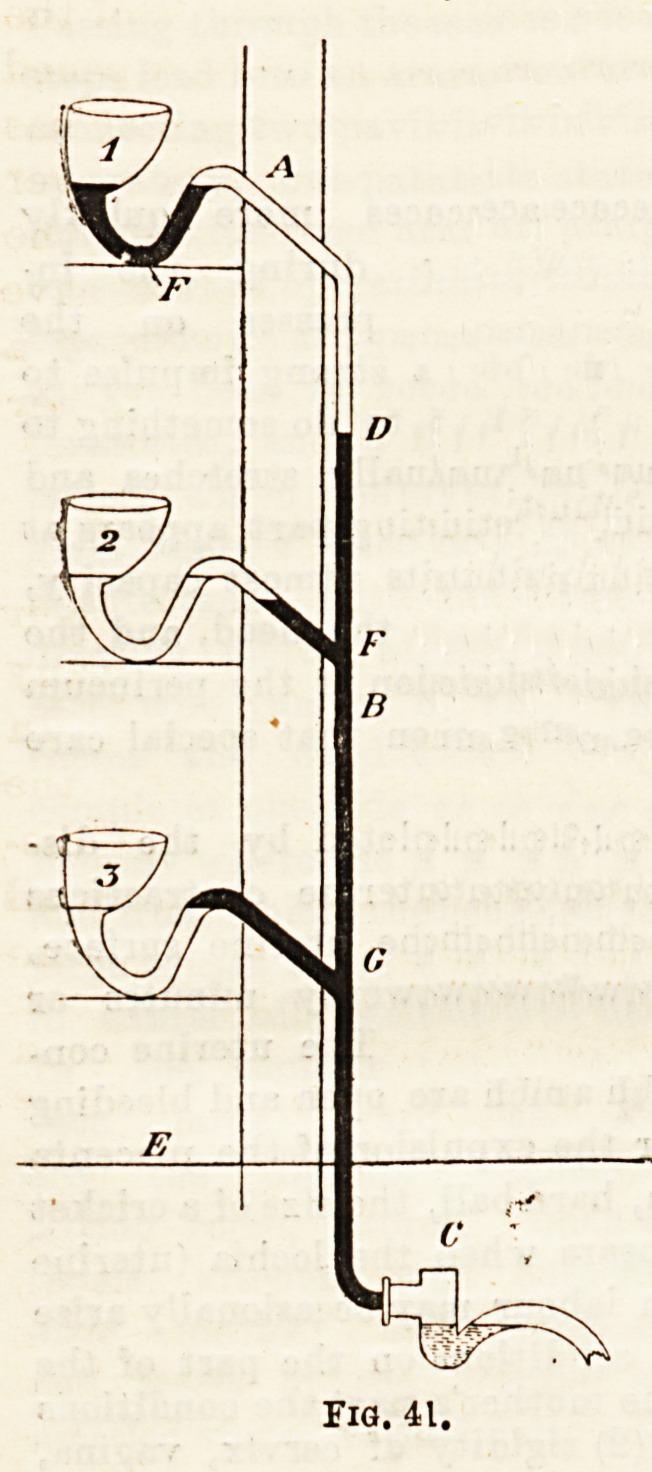


**Fig. 42. f2:**
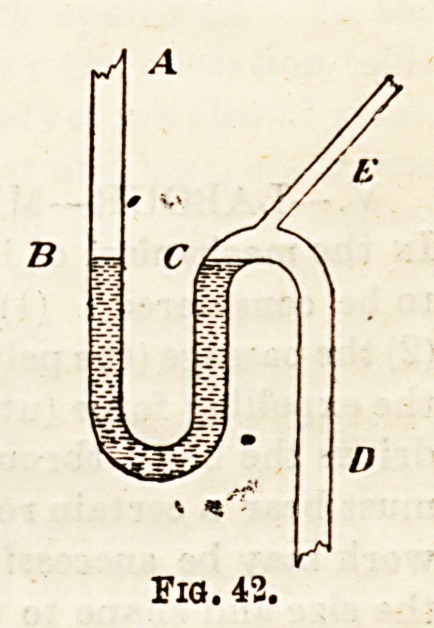


**Fig. 43 Buchan's Intercepting Ventilating Trap. f3:**
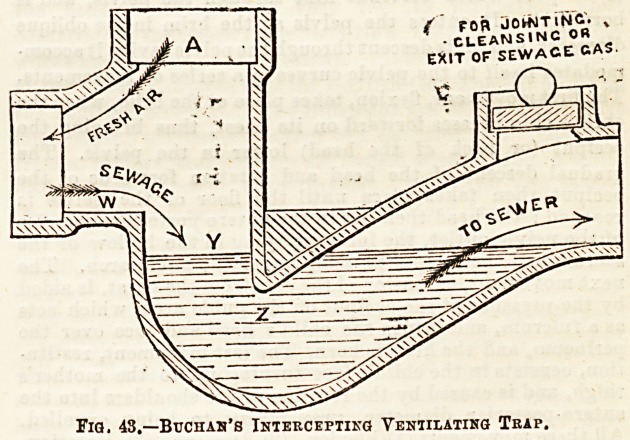


**Fig. 44. Buchan's Trap with Ventilating Eye. f4:**